# Two Divergent Genetic Lineages within the Horned Passalus Beetle, *Odontotaenius disjunctus* (Coleoptera: Passalidae): An Emerging Model for Insect Behavior, Physiology, and Microbiome Research

**DOI:** 10.3390/insects10060159

**Published:** 2019-06-04

**Authors:** Ryan C. Garrick, Trey Dickinson, Dana K. Reppel, Rachel N. Yi

**Affiliations:** Department of Biology, University of Mississippi, Oxford, MS 38677, USA; rdicki@lsuhsc.edu (T.D.); karenreppel@gmail.com (D.K.R.); rnyi1121@gmail.com (R.N.Y.)

**Keywords:** cryptic diversity, mitochondrial DNA, *Odontotaenius disjunctus*, southern Appalachian Mountains, saproxylic, subsocial

## Abstract

The horned passalus (*Odontotaenius disjunctus*) is one of the most extensively studied saproxylic beetles in the eastern United States. For several decades this species has been the subject of investigations into the behaviors associated with subsociality as well as physiological responses to stress, and, most recently, the composition of its gut microbiome has been closely examined. However, no published study to date has characterized this beetle’s broad-scale population genetic structure. Here, we conducted intensive geographic sampling throughout the southern Appalachian Mountains and surrounding areas and then assessed mitochondrial DNA (mtDNA) sequence variation among individuals. Unexpectedly, we discovered two divergent, yet broadly sympatric, mtDNA clades. Indeed, the magnitude of divergence between- vs. within-clades ranged from 5.9 to 7.5×, depending on the dataset under consideration, and members of the two lineages were often syntopic (i.e., found in the same rotting log). Given the potential implications for past and future studies on behavior, physiology, and the gut microbiome, we developed a simple cost-efficient molecular assay (i.e., polymerase chain reaction restriction fragment length polymorphism; PCR-RFLP) to rapidly determine mtDNA clade membership of *O. disjunctus* individuals. We suggest that the evolutionary processes that gave rise to the emergence and persistence of divergent sympatric lineages reported here warrant investigation, as this type of spatial-genetic pattern appears to be rare among southern Appalachian forest invertebrates.

## 1. Introduction

The horned passalus, *Odontotaenius disjunctus* Illiger (Coleoptera: Passalidae), is a subsocial wood-feeding beetle associated with decomposing hardwood logs. The species is broadly distributed across the eastern United States (U.S.), and can be locally very abundant in southern states [1]. Adults are large (3–4 cm long) and easily identified, in part because the only other native U.S. *Odontotaenius* species is endemic to a small region of south-central Florida [2]. They rarely fly [3], can easily be kept in captivity, and live for two years or more. These characteristics facilitate collection, direct observation, and experimental manipulation. Accordingly, *O. disjunctus* has been widely used in research, such as studies of life history, ecology, and larval development [4].

Investigations into the causes and consequences of subsociality have used *O. disjunctus* as a model for understanding a number of associated behaviors. Briefly, parents form a monogamous mate pair (but see [5]) and both of them provide long-term brood care, including activities such as excavation and maintenance of galleries and a nest, translocation of eggs, construction and repair of pupal cases, and provision of finely chewed pulp for young to feed on [1]. Parent-offspring cooperation in raising young, where siblings of different ages remain together in the same gallery system and the elder ones (i.e., freshly emerged teneral adults) contribute to the repair of pupal cases of the younger cohort, has been well-documented [6]. Other behaviors, such as aggression, movement, and territoriality, have also been studied intensively (Table 1). Indeed, adults and larvae are capable of acoustic communication via stridulation, with as many as 14 different signals identified—the most for any passalid beetle [7]. Furthermore, physiological research has used *O. disjunctus* as a model insect for investigations of several stress responses (e.g., dehydration, thermal tolerance, and parasite load), strength (pulling and lifting), respiration, as well as some interactions among these (Table 1).

Aside from the species’ suitability for a suite of investigations into insect behavior and physiology, several other biological characteristics of *O. disjunctus* have attracted research attention. For instance, to digest dead wood these beetles rely on a diverse array of microbes, including obligate endosymbionts that can assimilate xylose or provision essential amino acids or elements that are otherwise largely unavailable, and so the composition of their gut microbiome has begun to be characterized (Table 1). Ecologically, *O. disjunctus* is an important ecosystem service provider owing to its contribution to wood decomposition, and, by extension, nutrient cycling and soil formation processes in forests [33,34].

Taken together, *O. disjunctus* may be among the most extensively studied non-pest saproxylic beetles in the eastern U.S. However, to date there has been no published work on the species’ broad-scale genetic structure. Indeed, given that *O. disjunctus* individuals have poor dispersal (e.g., walking is the primary mode of dispersal and typical colonization distances may be <15 m; [10]), population differentiation seems likely. Here we used intensive geographic sampling followed by analysis of mitochondrial DNA (mtDNA) sequence data to characterize overall levels of polymorphism, assess spatial-genetic structure, and quantify the magnitude of lineage divergence. In addition to identifying the genetic structure among populations of *O. disjunctus*, we also developed a cost- and time-efficient molecular assay that would separate distinct mtDNA lineages.

## 2. Materials and Methods

### 2.1. Sampling

Between 2012 and 2017, *O. disjunctus* adults and/or larvae were sampled from intermediate- to late-stage rotting logs at 30 sites across five states spanning the southern Appalachians Mountains (110 individuals from 32 logs), as well as from six sites in Mississippi (15 individuals from seven logs; i.e., a total of 125 individuals from 39 logs, with an average of 3.2 beetles sampled per rotting log; see Table 2). We also integrated data from Swanson [35], who sampled four sites in the southern Appalachians (15 individuals from 15 logs) and six sites in Mississippi (28 individuals from 28 logs; Table 2). Ultimately, the present study was based on a total of 168 individuals.

### 2.2. Molecular Datasets

One leg was removed from each *O. disjunctus* specimen sampled in the present study (125 individuals), and genomic DNA was extracted from it using a DNeasy Blood and Tissue Kit (Qiagen, Valencia, CA, USA), following the manufacturer’s recommendations. For each individual, a fragment of the mitochondrial (mtDNA) *cytochrome oxidase subunit I* (*COI*) gene was amplified via polymerase chain reaction (PCR) using one of the following primer pair combinations: LCO-1490 and HCO-2198 [36], or LCO-1490 and OdCo1-R (5′-TGCGTAGATTATTCCTAATGC-3′; this study). A fragment of the mtDNA *cytochrome oxidase subunit II* (*COII*) gene was also amplified using primers developed here (OdCo2-F: 5′-AAAGCARTNGGACAYCAATG-3, and OdCo2-R: 5′-CATATSTTCAGTATCATT-3′). Amplifications were performed in 10 μL volumes (or multiples thereof) comprised of 2.0 μL 5× PCR buffer (Promega, Madison, WI, USA), 0.8 μL MgCl_2_ (25 mM, Promega), 1.6 μL dNTPs (1.25 mM, Promega), 0.5 μL bovine serum albumin (10 mg/mL, New England BioLabs; NEB, Ipswich, MA, USA), 3.0 μL dH_2_O, 0.5 μL of each primer, 0.1 μL Go-*Taq* (5 U/μL, Promega), and 1.0 μL of genomic DNA. The following PCR profile was used: 95 °C for 2 min (1 cycle), 95 °C for 30 s, 54 °C (*COI*) or 48 °C (*COII*) for 30 s, 72 °C for 1 min (35 cycles), and a final extension of 72 °C for 2 min (1 cycle). Products were viewed following agarose gel electrophoresis, purified using ExoSAP-IT (Affymetrix, Santa Clara, CA, USA), and sequenced on an Applied Biosystems 3730× Genetic Analyzer at Yale University’s DNA Analysis Facility on Science Hill. Sequences were edited and aligned in MEGA v6.06 [37].

Given that *COI* and *COII* genes are effectively part of the same locus owing to the non-recombining nature of animal mtDNA, aligned sequences (722-bp for *COI*, and 347-bp for *COII*; sequences of each unique haplotype are available from GenBank under accession numbers MK959501–MK959511 and MK959512–MK959519, respectively) were concatenated (1069-bp) for each individual. Of the 125 field-collected beetles included in the present study, most had complete genetic data (i.e., only 14 individuals were missing *COII*). Collectively, we refer to this as the “Garrick et al. dataset” herein. Additional *O. disjunctus COI* sequences were obtained from NCBI’s GenBank database (accession numbers DQ028959–DQ028983; 1097-bp alignment). Swanson [35] provided associated population-based haplotype frequency information and geographic coordinates, thus enabling geo-referenced *COI* sequence data for 43 additional individuals to be recreated. We refer to this as the “Swanson dataset” herein. There was partial overlap (441-bp) between the regions of *COI* sequenced here versus those sequenced by Swanson [35], and this provided an opportunity to integrate the two datasets. To do this, the Swanson dataset was trimmed to the homologous 441-bp, aligned against the Garrick et al. dataset, and missing data positions were represented using the IUPAC ambiguity character “N”. This yielded a 1069-bp alignment containing 168 individuals, which we refer to as the “combined dataset” herein.

### 2.3. Analyses

To characterize overall levels of polymorphism, for each of the three mtDNA datasets (see above) we calculated the number of segregating sites (*S*), number of parsimony-informative sites (*S*_pi_) and number of haplotypes (*N*_hap_), using MEGA and DNASP v5.10 [38]. To assess broad scale spatial-genetic structure in *O. disjunctus*, phylogenetic relationships among non-redundant mtDNA haplotypes in the combined dataset were estimated using maximum-likelihood, implemented in MEGA. We first identified the best-fit substitution model (i.e., HYK + I) using jModelTest 2 [39], and then performed tree searches using a maximum parsimony starting tree, empirical base frequencies, and extensive subtree-pruning-regrafting branch swapping. To assess node support, we used 1000 bootstrap replicates. Well-supported clades were identified visually, and the geographic distributions of their members were then mapped. To understand the magnitude of lineage divergence, two clade-based summary statistics were calculated in DNASP: the mean proportion of nucleotide differences among sequences within (*P*_within_) and between (*P*_between_) clades. The ratio of *P*_between_:*P*_within_ was also calculated. For comparison with the combined dataset, these statistics were recalculated separately for the Garrick et al. and Swanson datasets.

### 2.4. PCR Restriction Fragment Length Polymorphism (PCR-RFLP) Assay

Due to the discovery of two sympatric mtDNA lineages (see Results and Discussion), a cost- and time-efficient assay to determine mtDNA clade membership of *O. disjunctus* individuals was developed. Briefly, following Garrick et al. [40], clade-specific consensus sequences were generated from the complete dataset haplotype alignment, and then fixed nucleotide differences were identified. Next, NEBcutter v2.0 [41] was used to determine which of these could be resolved using commercially available restriction enzymes. Finally, we designed primers to amplify a short region, containing diagnostic differences, that produced interpretable banding patterns following restriction enzyme digestion and electrophoretic separation of fragments. Ultimately, a 244-bp fragment of the *COI* gene was targeted, using primers OdCo1rflp-F: 5′-TCTTCAATTATAAATATACGAAC-3′ and OdCo1rflp-R: 5′-TTAAAATGTAAACTTCAGGATGTCC-3′. Amplifications were performed in 10 μL volumes (or multiples thereof) with the same reaction mixture described above (see 2.2. *Molecular Datasets*), using the following PCR profile: 95 °C for 2 min (1 cycle), 95 °C for 30 s, 50 °C for 30 s, 72 °C for 30 s (35 cycles), and a final extension of 72 °C for 2 min (1 cycle). Amplified products were digested using one of two alternative approaches: (1) a single enzyme (*TaqI*) assay, or (2) a double digest (*BsaI* + *DdeI*) assay. All restriction digestions were performed in 25 μL reaction volumes. For the single enzyme assay, each reaction contained 15.0 μL dH_2_O, 2.5 μL of CutSmart buffer (NEB), 0.5 μL *TaqI* (5′-T/CGA-3′, 20 U/μL, NEB), and 7 μL of PCR product, and was incubated at 65 °C overnight. For the double digest assay, each reaction contained 14.5 μL dH_2_O, 2.5 μL of CutSmart buffer (NEB), 0.5 μL *BsaI* (5′-GGTCTCN/-3′, 20 U/μL, NEB), 0.5 μL *DdeI* (5′-C/TNAG-3′, 10 U/μL, NEB), and 7 μL of PCR product, and was incubated at 37 °C overnight. Electrophoretic separation of fragments was performed using 2% agarose gels with GelRed nucleic acid stain (Phenix Research Products, Asheville, NC, USA), and 1× TBE buffer. A 100-bp ladder (as well as undigested PCR product) was included, and gels were run for ~1.5 h at 80 V, 50 mA. Expected fragment sizes for two alternative PCR-RFLP assays are reported in Table 3.

## 3. Results and Discussion

**Figure 1:**
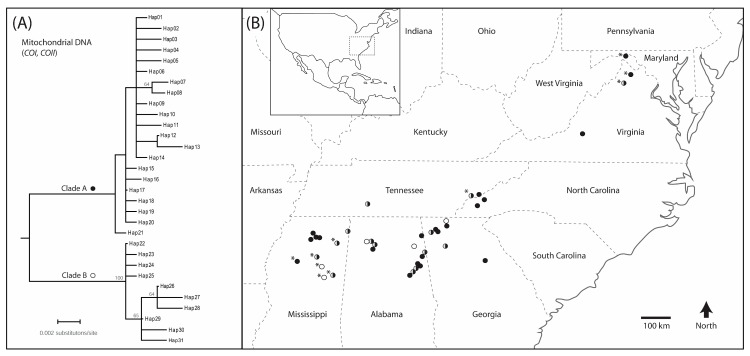
(**A**) Midpoint-rooted maximum-likelihood phylogenetic tree showing relationships among 31 non-redundant mtDNA haplotypes contained within the combined dataset. Numbers on nodes are bootstrap values (only those ≥ 50% are shown). Names of haplotypes are arbitrary; (**B**) Map showing the spatial distribution of 46 *Odontotaenius disjunctus* sampling sites (circles) across the southern Appalachian Mountains and surrounding areas, including northern Mississippi (see Table 2 for coordinates, and sample sizes per clade per site; asterisks indicate sites sampled by Swanson [35]). Different shading is used to represent the spatial distributions of clade A (solid) and clade B (open). Wherever members of both clades were sampled from the same site, the symbol is half solid and half open. Inset: map showing location of study area within the continental U.S. (dashed box).

Mitochondrial *COI* and *COII* sequences generated for *O. disjunctus* in the present study had A + T-biased nucleotide composition and open reading frames when translated to amino acids, consistent with expectations for “true” mtDNA as opposed to nuclear-mitochondrial pseudogenes [42]. Overall levels of polymorphism were moderate, with 20–31 unique haplotypes and 41–48 segregating sites (of which 28–31 were parsimony-informative), depending on the dataset under consideration (Table 4). Maximum-likelihood phylogenetic analyses of 31 non-redundant mtDNA haplotypes contained within the combined dataset identified two divergent and well-supported major lineages within *O. disjunctus* (clades A and B herein; Figure 1A). Overall, 123 individuals (73%) from 40 sites were members of clade A, whereas 45 individuals (27%) from 19 sites represented clade B (Table 4), with 21 versus 10 haplotypes in each clade, respectively. The spatial distributions of the two genetic lineages were broadly sympatric (Figure 1B). Indeed, representatives of both genetic lineages were present in 10 out of 27 rotting logs (37%; see Table 2) from which multiple individuals were sampled in the present study (since Swanson [35] collected one beetle per log, the frequency of syntopy based on samples from that study could not be assessed).

Divergent mtDNA lineages have been documented within numerous species of forest invertebrates from the southern Appalachian Mountains and surrounding areas, but in most cases, spatial distributions are allopatric. For example, the wood-feeding cockroach, *Cryptocercus punctulatus* (Blattodea: Cryptocercidae), is comprised of five mtDNA clades [43,44], and, although three of these occur in close proximity in the Great Smoky Mountains, they are locally non-overlapping (but see Garrick [45] for an anomaly). Similarly, the leaf-litter-dwelling flightless weevil, *Eurhoptus pyriformis* (Coleoptera: Curculionidae), is comprised of at least two lineages that show a spatial-genetic discontinuity in the vicinity of the Smoky Mountains [46]. Abrupt mtDNA-based phylogeographic breaks also occur in predatory arthropods, such as the spiders, *Hypochilus pococki* and *H. gertschi* (Araneae: Hypochilidae) [47], and the opilion, *Fumontana deprehendor* (Opiliones: Triaenonychidae) [48]. Some reports of parapatric distributions of mtDNA lineages also exist, for example, the millipede, *Narceus americanus* (Spirobolida: Spirobolidae) [49], the opilion, *Sabacon cavicolens* (Opiliones: Sabaconidae) [50], and the centipede, *Scolopocryptops sexspinosus* (Scolopendromorpha: Scolopocryptopidae) [51]. However, to our knowledge, broad sympatry—as seen in *O. disjunctus*—has not previously been described.

Comparison of mtDNA *COI* sequence differences between versus within distinct lineages has been proposed as a mechanism for recognizing the existence of cryptic species and accelerating the discovery of new species [52]. Based on a large avian dataset for which species boundaries were well-known, owing to extensive study using a combination of morphometrics, behavioral ecology, vocalization, and color pattern data, Hebert et al. [53] concluded that when the ratio of between-lineage versus mean within-lineage *COI* sequence divergence was at least 10×, then species-level (cf. population-level) status of those lineages could be assigned. For *O. disjunctus*, we found that the ratio of the proportion of nucleotide differences between- versus within-clades ranged from 5.9 to 7.5×, depending on the dataset under consideration (Table 4; note that when we recalculated these ratios using Kimura-2-parameter distances, as used by Hebert et al. [53], very similar outcomes were obtained). Thus, according to the “10× rule” criterion, at present we do not see a compelling reason to suspect the two *O. disjunctus* clades are indicative of cryptic species. That said, we do recognize that Hickerson et al. [54] demonstrated that, under several realistic circumstances, Hebert et al.’s [53] threshold can be too stringent and therefore overlooks a lot of species-level diversity. Nonetheless, in the absence of independent datasets that show concordant delimitation of lineages (e.g., [55]), we cautiously consider *O. disjunctus* to be a single species. Indeed, there are well-known limitations even with inferring population structure using a single locus, and so we advocate for a follow-up work that will re-assess the present inferences.

Notwithstanding our speculation that population-level processes (e.g., demographic history; see [56]) likely generated and maintain the two divergent, yet sympatric, mtDNA-based lineages reported here, there may, nonetheless, be consequential impacts on other fields of research for which *O. disjunctus* is used as model species (see Introduction, and Table 1). For example, deeply divergent intraspecific mtDNA clades can be indicative of corresponding differences in nuclear DNA regions (e.g., [57]). Furthermore, direct interactions between mitochondrial genes that encode for oxidative phosphorylation and nuclear genes that encode structural and other components of the mitochondrion are functionally important [58], and, as such, co-adapted mitonuclear gene complexes can inhibit gene flow among populations (e.g., [59]). While it remains to be determined whether nuclear DNA sequences from *O. disjunctus* reflect the mtDNA patterns reported here, we advocate for explicit reporting of which mtDNA clade(s) is/are the subjects of future studies focusing on this species. Indeed, it is quite plausible that members of both clades were inadvertently included in previous studies on behavior, physiology, and the gut microbiome. For instance, all of those studies used field-collected beetles, usually sampled from several different rotting logs, and often assessed ≥ 20 individuals (Table 1). Accordingly, there is no reason to expect that the focal individuals were members of the same family and thus possessed the same or similar mtDNA haplotypes. Furthermore, although only four of the 11 eastern U.S. states from which these previous studies obtained specimens were included in the present study, given the broadly sympatric distributions of clades A and B (Figure 1B), we suggest that this previously unrecognized (and potentially confounding) variable may be relevant to many studies of *O. disjunctus*.

In light of the value of a rapid means for determining mtDNA clade membership of *O. disjunctus* individuals, we developed two alternative PCR-RFLP assays that produced easily distinguishable banding patterns (Table 3). Furthermore, both assays were empirically validated (i.e., several *O. disjunctus* individuals of known clade membership were genotyped and there were no unexpected outcomes; see Supplementary Material, Figure S1), suggesting that they could become broadly useful. However, as a precaution, we do suggest that some additional cross-validation (e.g., via direct sequencing) may be warranted if specimens are collected outside of the geographic range sampled in the present study.

## 4. Conclusions

Insights into the evolutionary processes that gave rise to two mtDNA lineages within *O. disjunctus* are beyond the scope of this study, but demand exploration. Such information can be gained via phylogeographic analysis of geo-referenced multi-locus sequence data from population samples of the beetles, and potentially also by comparative analyses of their symbionts or other ecologically co-associated taxa (e.g., [44,60,61,62]). In the former context, the utility of next-generation sequencing approaches for generating genome-wide nucleotide polymorphism data has already been demonstrated for this species (e.g., [8]), such that strong historical inferences should be achievable. However, for now, past and future studies on behavior, physiology, and the gut microbiome of *O. disjunctus* should benefit from new knowledge of the existence of divergent lineages, and the ability to rapidly determine mtDNA clade membership of individuals via the PCR-RFLP assays reported here.

## Figures and Tables

**Table 1:** Summary of published literature that used *O. disjunctus* as a model for insect behavior, physiology, and gut microbiome research. State abbreviations for geographic origins of specimens are as follows: Alabama (AL), Georgia (GA), Kansas (KS), Kentucky (KY), Louisiana (LA), Maryland (MD), Mississippi (MS), North Carolina (NC), Pennsylvania (PA), Southern Carolina (SC) and Virginia (VA); NR = not reported.

Biological Discipline	Research Focus	No. of Rotting Logs Sampled	No. of Individuals Used	Location/Origin of Specimens ^a^	Reference
Behavior	Brood care, delayed juvenile dispersal	6	92	Wolfe Co., KY	[8]
	Brood care, extra-pair paternity	6	88	Wolfe Co., KY	[5]
	Brood care, infanticide	NR	291	Williamsburg, VA	[9]
	Movement behavior, habitat edge effects	>1	76	Baton Rouge, LA	[10]
	Movement behavior, territory size	many	many	multiple parishes, LA	[11]
	Movement behavior, local density	many	many	Baton Rouge, LA	[12]
	Movement behavior, nuptial flights	NR	12–15	Oktibbeha Co., MS	[3]
	Aggression, resident/intruder interactions	24	24	Shelby Co., AL	[13]
	Aggression, fighting ability vs. parasite load	>1	192	Clarke Co., GA	[14]
	Anti-predator behavior, deterrence	>1	28	Prince Georges Co., MD	[15]
Physiology	Thermal tolerance, water loss & dehydration	NR	24	Piedmont region, NC ^a^	[16]
	Thermal tolerance, overwintering stress	NR	40	Forsyth Co., NC	[17]
	Respiration, tracheal system compression	NR	5	Piedmont region, NC ^a^	[18]
	Heart rate, stress response vs. parasite load	>1	99	Clarke Co., GA	[19]
	Heart rate, stress response vs. parasite load	>1	17–77	Clarke Co., GA	[20]
	Strength, pulling force vs. size & gender	>1	41	Clarke Co., GA	[21]
	Strength, pulling force vs. parasite load	>2	92	Clarke Co. & Chatham Co., GA	[22]
	Strength, lifting capacity vs. mild stress	>1	20	Clarke Co. & Chatham Co., GA	[23]
	Strength, lifting capacity vs. parasite load	>1	40	Clarke Co., GA	[24]
Gut microbiome	Xylose-fermenting yeast	>5	NR	Burke Co. & Clarke Co., GA; Baton Rouge, LA; Chester Co., PA; Orangeburg Co., SC	[25,26]
	Xylose-fermenting yeast	>10	>300	Southeastern, Hammond, LA	[27]
	Xylose-fermenting yeast	>1	NR	Southeastern, Hammond, LA	[28]
	Discovery & description of a new genus of yeast	>1	NR	Douglas Co. KS	[29]
	Compartmentalization and N-fixation (bacteria & archaea)	1	8	Baton Rouge, LA	[30]
	Compartmentalization and microbial functional assembly	NR	39	Baton Rouge, LA	[31]
	Microbiome change over time (bacteria, fungi, & protists)	>1	NR	Baton Rouge, LA	[32]

^a^ Beetles were purchased from Carolina Biological Supply Company, which provides field-collected specimens from the Piedmont region in central NC.

**Table 2:** Geographic locations from which *O. disjunctus* beetles were sampled, and their membership in mitochondrial DNA clades A or B. Abbreviations associated with region names are: Mountain(s), Mtn; National Forest, NF; National Park, NP; National Wildlife Refuge, NWR; Parkway, Pkwy; State Park, SP; University of Mississippi Field Station, UMFS; and Wildlife Management Area, WMA.

State	Region	Site ID	No. of Logs	Longitude	Latitude	No. of Clade A Individuals	No. of Clade B Individuals
Alabama	Little River Canyon Nature Preserve	A134	1	34.45540	−85.58357	3	0
	Bankhead NF	A130	1	34.29811	−87.38140	1	5
		C10	1	34.28235	−87.39905	0	1
		A133	1	34.17659	−87.27680	6	1
		C13	1	34.10336	−87.32465	1	0
		C15	1	34.10230	−87.32407	1	0
		C16	1	34.10151	−87.32454	1	0
	Shinbone Ridge Road	A138	1	34.14676	−85.84679	0	2
	Talladega NF	A137 ^a^	2	33.96340	−85.45730	7	2
		A41	1	33.91858	−85.49764	2	0
		A12	1	33.57157	−85.69391	2	0
		A13	1	33.56059	−85.70074	6	0
		A117	1	33.47105	−85.80658	4	2
		A124	1	33.40451	−85.87318	7	0
		A15 ^b^	2	33.39762	−85.88391	7	2
		A126	1	33.36136	−85.93017	5	0
Georgia	W-Chattahoochee NF	A18	1	34.87866	−84.71137	0	1
		A154	1	34.75933	−84.69117	3	0
	Johns Mtn WMA	A04	1	34.57297	−85.06536	1	0
		A153	1	34.57281	−85.06541	1	0
		A152	1	34.56806	−85.07185	1	0
		A10	1	34.56416	−85.24043	2	3
	Red Top Mtn SP	A140	1	34.15014	−84.71650	1	1
	Oconee NF	A150	2	33.72088	−83.29258	4	0
Mississippi	Tishomingo SP	A86 ^c^	2	34.60502	−88.19299	2	3
	Holly Springs NF	M01	1	34.50676	−89.44006	1	0
		M05	1	34.50665	−89.44013	1	0
	UMFS	FS03	1	34.42800	−89.38657	3	0
		M14	1	34.42396	−89.38293	2	0
	Whirlpool Trail	M13	1	34.34670	−89.55107	3	0
	Tombigbee SP	M-5 ^d^	3	34.23167	−88.61667	2	1
	County Road 484	M-4 ^d^	4	33.83333	−89.33333	2	2
	Malmaison State WMA	M-6 ^d^	1	33.68667	−90.04333	1	0
	Natchez Trace Pkwy	M-3 ^d^	4	33.52833	−89.16667	0	4
	Noxubee NWR	M-1 ^d^	9	33.29167	−88.77833	5	4
	Tombigbee NF	M-2 ^d^	7	33.20667	−89.07500	0	7
North Carolina	Great Smoky Mtn NP	A156	1	35.52144	−83.30981	3	0
	Nantahala NF	A92	1	35.32969	−83.59187	14	0
Tennessee	Great Smoky Mtn NP	A159	1	35.66272	−83.52245	2	0
	Rich Mtn. Loop Trail	T-1 ^d^	3	35.61667	−83.79833	2	1
	Natchez Trace Pkwy	A73	1	35.39384	−87.52677	1	2
Virginia	Tuleyries Lane	V-2 ^d^	6	39.06167	−78.07500	6	0
	Shenandoah River SP	V-1 ^d^	4	38.84167	−78.31000	3	1
	Jefferson NF	A53	1	37.40599	−79.80718	2	0
West Virginia	Cacapon Resort SP	V-3 ^d^	2	39.59000	−78.27667	2	0

^a^ Multiple individuals were sampled from one log, and both lineages were represented in that log; ^b^ multiple individuals were sampled from both logs, but both lineages were represented in only one log; ^c^ multiple individuals were sampled from both logs, and both lineages were represented in each log; and ^d^ Collections and associated mitochondrial *COI* sequence data extracted from Swanson [35].

**Table 3:** Expected fragment sizes (bp) for two alternative PCR-RFLP assays that can be used to distinguish between two divergent genetic lineages of *O. disjunctus* based on initial PCR amplification of a 244-bp section of mtDNA *COI* (see Supplementary material, Figure S1). The single enzyme (*TaqI*) RFLP assay differentiates clade A from clade B by cutting either once or twice, respectively. The double digest RFLP assay cuts once in all cases, but at different diagnostic DNA polymorphisms (*BsaI* cuts clade A haplotypes, whereas *DdeI* cuts clade B haplotypes).

PCR-RFLP Assay/MtDNA Genetic Lineage	*TaqI* Single Digest	*BsaI* + *DdeI* Double Digest
Clade A	103/141	68/176
Clade B	23/80/141	108/136

**Table 4:** Characteristics of mtDNA sequence data generated from *O. disjunctus* individuals sampled in the present study, a previous study by Swanson (2005), and a combined dataset. Summary statistics are as follows: number of haplotypes (*N*_hap_), number of segregating sites (*S*), number of parsimony-informative sites (*S*_pi_), and mean proportion of nucleotide differences among sequences within (*P*_within_) and between (*P*_between_) clades identified following phylogenetic tree estimation (see Figure 1). Ratio was calculated as *P*_between_ divided by *P*_within_ mean.

MtDNA Dataset/Statistic	Garrick et al. Dataset	Swanson Dataset	Combined Dataset
*N*_hap_	20	25	31
*S*	41	41	48
*S*_pi_	29	28	31
*P*_within_ clade A	0.0029	0.0034	0.0035
*P*_within_ clade B	0.0022	0.0028	0.0044
*P*_within_ mean	0.0026	0.0031	0.0040
*P*_between_	0.0194	0.0228	0.0233

## Supplementary Materials

The following is available online at https://www.mdpi.com/2075-4450/10/6/159/s1, Figure S1: Exemplar agarose gel showing banding patterns for *TaqI* and *BsaI* + *DdeI* PCR-RFLP assays.
