# Supplementary material for: Two Divergent Genetic Lineages within the Horned Passalus Beetle, Odontotaenius disjunctus (Coleoptera: Passalidae): An Emerging Model for Insect Behavior, Physiology, and Microbiome Research
Source: Insects. 2019 Jun 4;10(6):159. doi: 10.3390/insects10060159 (PMC6628224; doi:10.3390/insects10060159)
Supplement: Supplementary file 1 [file insects-10-00159-s001.pdf]

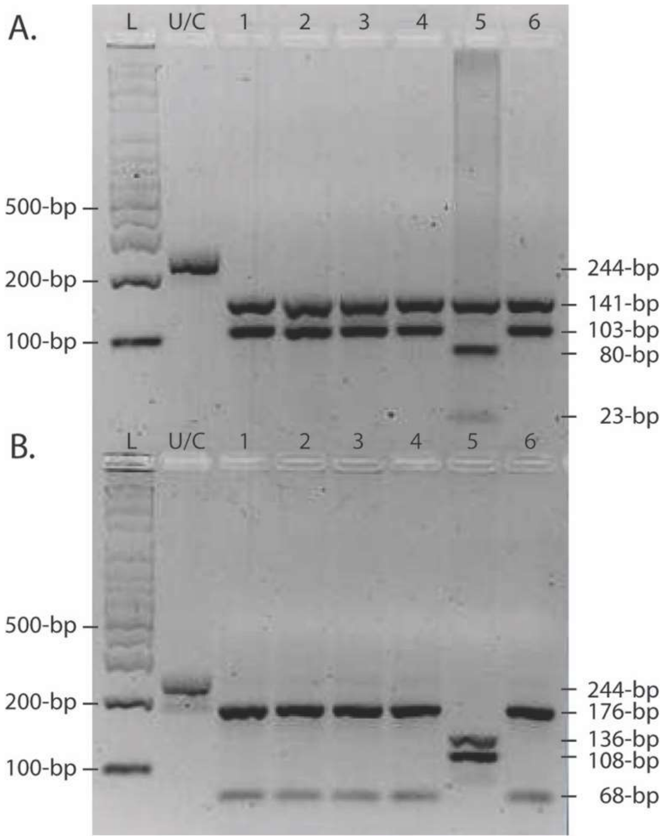

Figure S1. Exemplar agarose gel showing PCR-RFLP banding patterns for (A) the single enzyme (*TaqI*) assay, and (B) the double digest (*BsaI* + *DdeI*) assay, both of which can be used to distinguish between two *O. disjunctus* mtDNA clades. Lanes are labeled as follows: L, 100-bp ladder; U/C, uncut PCR product; 1–6: sample lanes that contain individuals of known clade membership (1–4 and 6 = clade A, 5 = clade B). Known fragment sizes (bp) derived from the ladder are labeled on the left, and expected fragment sizes are on the right.
